# Detecting Multi-Resolution Pedestrians Using Group Cost-Sensitive Boosting with Channel Features †

**DOI:** 10.3390/s19040780

**Published:** 2019-02-14

**Authors:** Chao Zhu, Xu-Cheng Yin

**Affiliations:** 1 School of Computer and Communication Engineering, University of Science and Technology Beijing, Beijing 100083, China; xuchengyin@ustb.edu.cn; 2 Beijing Key Laboratory of Knowledge Engineering for Materials Science, Beijing 100083, China

**Keywords:** pedestrian detection, multi-resolution, group cost-sensitive boosting

## Abstract

Significant progress has been achieved in the past few years for the challenging task of pedestrian detection. Nevertheless, a major bottleneck of existing state-of-the-art approaches lies in a great drop in performance with reducing resolutions of the detected targets. For the boosting-based detectors which are popular in pedestrian detection literature, a possible cause for this drop is that in their boosting training process, low-resolution samples, which are usually more difficult to be detected due to the missing details, are still treated equally importantly as high-resolution samples, resulting in the false negatives since they are more easily rejected in the early stages and can hardly be recovered in the late stages. To address this problem, we propose in this paper a robust multi-resolution detection approach with a novel group cost-sensitive boosting algorithm, which is derived from the standard AdaBoost algorithm to further explore different costs for different resolution groups of the samples in the boosting process, and to place greater emphasis on low-resolution groups in order to better handle the detection of multi-resolution targets. The effectiveness of the proposed approach is evaluated on the Caltech pedestrian benchmark and KAIST (Korea Advanced Institute of Science and Technology) multispectral pedestrian benchmark, and validated by its promising performance on different resolution-specific test sets of both benchmarks.

## 1. Introduction

**Figure 1:**
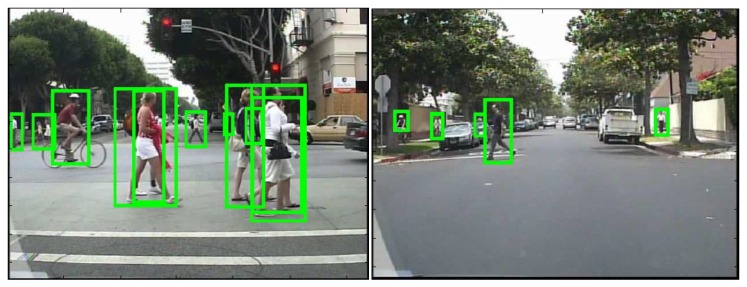
Example images and ground truth annotations in the Caltech pedestrian benchmark. Note that the resolutions of the pedestrians are in a wide range.

Object detection is a hot and challenging topic in the computer vision and multimedia community [1]. As an important task in this domain, pedestrian detection has received special interest because of its considerable applications in practice, such as video surveillance, crowd understanding, tracking, assistant driving, and robot navigation. Owing to lots of effort and many different detection approaches proposed in the literature, impressive progress has been achieved in the past few years. Nevertheless, it is still difficult to detect multi-resolution pedestrians in images and videos (as shown in Figure 1), and existing approaches suffer from their great performance drop with reducing resolution of the detected targets. For example, state-of-the-art detectors in the literature nowadays can achieve less than 1% of a mean miss rate for the detection of pedestrians taller than 80 pixels in the Caltech pedestrian benchmark [2], while the mean miss rate significantly increases to more than 50% for the detection of pedestrians who are 30–80 pixels high. Meanwhile, it is required to achieve robust detection of low-resolution targets in certain circumstances. For example, accurate detection of low-resolution pedestrians is very important in assistant driving systems so that necessary time can be provided to take the reactions.

Because of both high effectiveness and high efficiency, the boosting-based approaches are popular in pedestrian detection literature for detector training [2]. The main idea is to linearly train a series of weak classifiers and then combine them to construct a strong classifier. In the boosting process, each training sample is assigned with a weight which is used to calculate its corresponding classification cost, and is updated iteratively according to the classification results in each iteration so that the wrongly classified samples can be better emphasized. In the case of pedestrian detection, the truth is compared to the huge number of negative windows, where only small number of positive targets need to be detected. Therefore, the positive samples should possess greater weights during training so that a higher detection rate can be achieved. To that end, the researchers in the community have proposed several cost-sensitive boosting algorithms [3,4,5] where the false negatives are given more penalties than the false positives so that more importance is put on the positive samples. However, these algorithms are not optimal for multi-resolution detection, since they still treat all positive samples equally and ignore their intra-class variations. Due to the missing details of the appearances for low-resolution pedestrians, the features extracted from low-resolution samples are usually less discriminative than that from high-resolution ones, leading to the consequence that low-resolution pedestrians could be regraded as false negatives since they are more easily rejected during boosting in the early stages and can hardly be recovered in the late stages. Consequently, the trained detectors are possibly biased towards high-resolution pedestrians and leads to poorer performance on low-resolution pedestrians.

In order to address this problem, we propose in this paper a new group cost-sensitive boosting algorithm for robust multi-resolution pedestrian detection. In particular, we integrate the proposed algorithm with two representative detection frameworks: Locally Decorrelated Channel Features (LDCF) [6] and Convolutional Channel Features (CCF) [7], and propose a multi-resolution LDCF approach and a multi-resolution CCF approach, respectively. The proposed approaches can explore different costs for different resolution groups of the samples in the boosting process, and put greater importance on low-resolution pedestrians in order to better handle the detection of multi-resolution targets.

The main contributions of this work can be summarized as follows:

- Different from the existing approaches that treat all positive samples equally and ignore their intra-class variations in the boosting process, we propose a new group cost-sensitive boosting algorithm to further explore different costs for different resolution groups in positive set, so that low-resolution pedestrians can be better emphasized in the training process, leading to better detection in multi-resolution cases.
- We integrate the proposed algorithm with two representative detection frameworks: one is based on the classical hand-crafted features (LDCF) and the other is based on the popular deep-learning features (CCF), so that its effectiveness and generalization capability can be better validated.
- We evaluate the proposed approaches on two challenging pedestrian detection benchmarks (the Caltech pedestrian dataset and the KAIST multispectral pedestrian dataset), and the results show their promising performances compared to other state-of-the-art approaches on different resolution-specific test sets.

A preliminary version of this work appeared in [8]. This paper significantly extends it in the following ways. Firstly, we only consider the case of two resolution groups in the proposed boosting algorithm in [8], while in this paper we extend it to a generalized case of *N* resolution groups, so that the proposed approach can be more easily applied in other specific problems. Secondly, besides the LDCF detection framework as the baseline in [8], we also integrate in this paper the proposed algorithm with the CCF detection framework, which is based on the popular deep-learning features, in order to further improve detection performance and better validate its effectiveness. Thirdly, we add in the Appendix A of this paper a detailed proof of the key solution in the proposed group cost-sensitive boosting algorithm. Finally, besides the Caltech pedestrian benchmark used in [8], we conduct more experimental evaluation on an additional KAIST (Korea Advanced Institute of Science and Technology) multispectral pedestrian benchmark to validate the effectiveness of the proposed approach more extensively.

The remainder of the paper is organized as follows. After reviewing the related work in Section 2, we present the details of the proposed group cost-sensitive boosting algorithm for multi-resolution detection in Section 3. Then Section 4 reports the experimental evaluation for the effectiveness of the proposed approach. Finally, we conclude the paper in Section 5.

## 2. Related Work

Pedestrian detection has attracted attention for decades and has achieved impressive progress thanks to many effective detection techniques proposed in the literature [2]. However, only limited attention has been paid in the literature [9,10,11,12] on the problem of multi-resolution detection. In [9], a multi-resolution model of pedestrians was proposed consisting of a rigid HOG (Histogram of Oriented Gradient) template used to score low-resolution instances and a deformable part-based model used to score high-resolution instances. The motivation lies in that low-resolution instances usually lose lots of visual detail due to their small scales, meaning that a rigid HOG template is sufficient to characterize their global appearance features. On the contrary, high-resolution instances contain more detailed information; thus, a more complex part-based model could be applied to capture more detailed features from different parts and to improve accuracy. In [10], the authors propose training multiple models for different scales to perform multi-scale detection. Different from the traditional approaches that train *N* models, each for an individual scale, which is highly computational-cost centered, the key idea of the authors is to reduce the number of models for feature computation by a factor *K* and to resize images at training time instead of at test time. The computed *N*/*K* models will be used at test time to approximate the models in the remaining *N-N*/*K* scales. The main focus of this work is on detection speedup more than on detection accuracy. In [11], the authors propose an approach of using scale-independent features and one single classifier for all pedestrian scales. For image representation, HOG, LBP (Local Binary Patterns), and LUV color descriptors are adopted and the codebook maps are calculated based on the bag-of-visual-words model of each descriptor. These maps are then decomposed into channels for each individual word to obtain the proposed word channels feature. For multi-scale detection, one single classifier is trained based on the scale-independent classification features computed on word channels, and is applied on all sliding window scales. The authors in [12] take pedestrian detection in different resolutions as different but related problems, and propose a multi-task model to jointly consider their relations and differences. They first map pedestrians in different resolutions to a common space via resolution-aware transformations, and then train a shared detector in that space to perform multi-scale pedestrian detection. Nevertheless, this method relies on the deformable part-based model, and thus has relatively high computational complexity.

**Figure 2:**
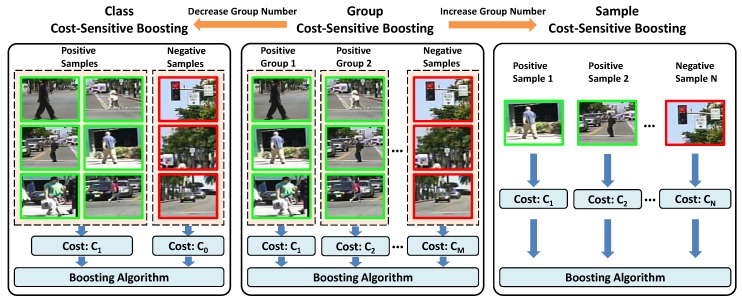
Comparison of different cost-sensitive boosting strategies.

In order to achieve more efficient detection, the boosting-based approaches are popular for training detectors. Several cost-sensitive boosting algorithms have been proposed in the literature to address the problem of sample imbalance, and can be classified into two categories: one is class cost-sensitive boosting (denoted as CCS boosting) such as Asymmetric-AdaBoost [3], AdaCost [13], CSB0-CSB2 [14], AdaC1-AdaC3 [4], and cost-sensitive boosting [5]; and the other is sample cost-sensitive boosting (denoted as SCS boosting) [15]. In CCS boosting, the cost is determined by the type of classification errors, i.e., misclassifying a sample into different classes will lead to different costs. In SCS boosting, the cost is determined by the samples, i.e., different samples will lead to different costs, no matter whether their types of classification errors are the same or not. Nevertheless, these two kinds of methods share the same main idea of putting more costs on the misclassified positive samples by modifying the weight update rules in boosting, so that false negatives are more penalized than false positives. However, although these algorithms distinguish positive samples from negative ones in the boosting process, they still ignore the possible variations inside the positive set. Different from these methods, our proposed approach is based on a new group of cost-sensitive boosting (denoted as GCS boosting) which explores different costs for different resolution groups in the positive set during boosting in order to better handle the detection in multi-resolution situations. Note that the proposed approach is related to both CCS boosting and SCS boosting, as shown in Figure 2, and can be considered as a generalized form of them. In the special case of a decreasing group number where all positive samples are treated as one group, GCS boosting will be simplified to CCS boosting, while in the special case of increasing group numbers to treat each positive sample as an individual group, GCS boosting will scale up to SCS boosting.

## 3. Multi-Resolution Detection via Group Cost-Sensitive Boosting with Channel Features

In this section, we present the details of the proposed multi-resolution detection approach with a new group cost-sensitive boosting algorithm, which is derived from the standard AdaBoost algorithm by further exploring different costs for different resolution groups of the samples in the boosting process, so that low-resolution groups can obtain greater importance and more emphasis in order to achieve better detection of multi-resolution targets.

### 3.1. Baseline Detection Frameworks

We consider in this paper two representative detection frameworks as a baseline: one is Locally Decorrelated Channel Features (LDCF) [6], which is based on the classical hand-crafted HOG and color features, and the other is Convolutional Channel Features (CCF) [7], which is based on the popular deep-learning features.

#### 3.1.1. Locally Decorrelated Channel Features (LDCF)

Given an input image, the LDCF approach calculates several image channels as a feature at first, where each image channel is a per-pixel feature map—in other words, the output pixels are calculated with their corresponding input pixels. Then, by applying a feature transform, the correlations in local image patches are removed. The idea is to replace the expensive oblique splits by the efficient orthogonal splits on locally decorrelated data in decision trees. In total, we calculated 10 feature channels, including one channel of the normalized gradient magnitude, six channels of the histogram of oriented gradients, and three channels of LUV color, and then applied four decorrelating filters for each channel, and finally obtained 40 locally decorrelated channels as features. To train detectors, we adopted the AdaBoost algorithm to train a certain number of decision trees on these channel features and then combined them to construct a strong classifier. More details of the LDCF approach can be referred to [6].

#### 3.1.2. Convolutional Channel Features (CCF)

The CCF approach generally has similar workflow to the traditional channel-feature-based approaches, like the aforementioned LDCF, in that it consists of two components: image feature extraction, and classifier learning via boosting. However, the main difference lies in that CCF takes advantage of the recently developed deep-learning techniques, and replaces the hand-crafted HOG and color features used in the conventional channel-feature-based approaches by the deep-learning-based convolutional features in order to obtain performance improvements by utilizing better image representations. For the feature extraction component, CCF extends multiple channel features to low-level feature maps transferred from the first few layers of a CNN model pre-trained on an ImageNet image dataset. For the classifier learning component, CCF trains an ensemble of decision trees in a boosting manner, with each node in decision trees dependent on one pixel value in the candidate feature maps. To perform the detection, the learned decision tree model is applied on dense image patches and the output of each decision tree is accumulated to get the final result. Specifically, the “conv3-3” layer in the VGG-16 model is adopted as the final feature representation, and a sliding window strategy is applied during detection. More details of the CCF approach is referred to in [7].

#### 3.1.3. Detection via AdaBoost

To facilitate understanding of the following description, a formal definition of the problem of detection via AdaBoost is given as follows: we first list in Table 1 the terms that will appear in the following equations, and describe how they are related to the multi-resolution pedestrian detection problem.

Given a number of samples ${\left\{(x_{i},y_{i})\right\}}_{i=1}^{n}$ for detection, where $x={\left(x_{1},\ldots ,x_{N}\right)}^{T}\in X=R^{N}$ is the feature representation of samples, and y∈Y={−1,1} is the class label of samples, a detector (or so-called binary classifier) is defined as a function *h* that can map each feature x to its corresponding class label *y*, and is usually implemented as follows:

(1) h(x)=sgn[f(x)]

where f(x) is a predictor, sgn[·] is the sign function which will be 1 if f(x)≥0, and will be −1 otherwise. If the detector can minimize the risk $E_{X,Y}\left[Loss\left(x,y\right)\right]$, where Loss(x,y) is a loss function to measure the classification error of the samples, then it will be considered as optimal. Recall that in the baseline LDCF and CCF approaches, the following loss function is adopted in the AdaBoost algorithm:

(2) $$Loss\left(x,y\right)=\left\{\begin{array}{cc} 0, & \mathrm{if}\;h(x)=y\\ 1, & \mathrm{if}\;h(x)\neq y \end{array}\right.$$

and a predictor f(x) is learned by linearly combining the weak learners as follows:

(3) $$f\left(x\right)=\sum_{m=1}^{M}\alpha_{m}g_{m}\left(x\right)$$

where $\alpha_{m}$ is a set of weights for different weak learners and $g_{m}\left(x\right)=\mathrm{sgn}\left[\phi_{m}\left(x\right)-t_{m}\right]$ is a set of decision stumps with $\phi_{m}\left(x\right)$ being a feature response and $t_{m}$ being a threshold.

Particularly, the predictor f(x) can be learned by the gradient descent with respect to the following exponential loss:

(4) $$E_{X,Y}\left[\exp\left(-yf\left(x\right)\right)\right]$$

and we iteratively select the weak learners so that the classification error is minimized at each iteration:

(5) $$g_{m}\left(x\right)=\arg\min_{g}\left(err_{\left(m\right)}\right)$$

where

(6) $$err_{\left(m\right)}=\sum_{i=1}^{n}\omega_{i}^{\left(m\right)}\left[1-I\left(y_{i}=g_{m}\left(x_{i}\right)\right)\right]$$

is the total classification error, and I(·) is an indicator function, as follows:

(7) $$I\left(y=a\right)=\left\{\begin{array}{cc} 1, & \mathrm{if}y=a\\ 0, & \mathrm{if}y\neq a \end{array}\right.$$

We calculated the weight of each weak learner as:

(8) $$\alpha_{m}=\frac{1}{2}\log\left(\frac{1-err_{\left(m\right)}}{err_{\left(m\right)}}\right)$$

and updated the weight $\omega_{i}^{\left(m\right)}$ of each sample so that at the next iteration, the importance of the wrongly classified samples was increased, and the importance of the correctly classified samples was decreased:

(9) $$\omega_{i}^{\left(m+1\right)}=\omega_{i}^{\left(m\right)}\exp\left(-y_{i}\alpha_{m}g_{m}\left(x_{i}\right)\right)$$

### 3.2. Group Cost-Sensitive Boosting Algorithm

Note that the loss function defined in Equation (2) is cost-insensitive because of the same costs of the false positives (y=−1,h(x)=1) and the false negatives (y=1,h(x)=−1) in this function. In order to deal with the multi-resolution detection in a better way, a new group cost-sensitive AdaBoost algorithm is proposed by exploring the different importance of the samples from different resolution groups so that low-resolution samples, which are usually harder to be detected, can have more emphasis in the boosting process.

#### 3.2.1. Group Cost-Sensitive Loss

In order to assign different importance to samples of different resolution, the positive samples were further divided into *N* groups (G1,G2,…,GN) according to their different resolutions (here we assume the groups are sorted in a resolution-ascending order, i.e., the samples in GN had larger resolution than the samples in GN−1). Then, a group cost-sensitive loss function was proposed as follows:

(10) $$Loss\left(x,y\right)=\left\{\begin{array}{cc} 0, & \mathrm{if}h(x)=y\\ C_{fp}, & \mathrm{if}y=-1,h(x)=1\\ C_{fn1}, & \mathrm{if}y=1,h(x_{G1})=-1\\ C_{fn2}, & \mathrm{if}y=1,h(x_{G2})=-1\\ \vdots & \vdots \\ C_{fnN}, & \mathrm{if}y=1,h(x_{\mathrm{GN}})=-1 \end{array}\right.$$

where $C_{*}>0$. In this loss function, different scenarios are respectively considered, including correct detections (h(x)=y), false positives (y=−1,h(x)=1), false negatives (miss detections) of samples in a resolution group G1 ($y=1,h(x_{G1})=-1$), false negatives of samples in a resolution group G2 ($y=1,h(x_{G2})=-1$), …, and false negatives of samples in a resolution group GN ($y=1,h(x_{GN})=-1$). Note that in the case of $C_{fn1}=C_{fn2}=\ldots =C_{fnN}$, this group cost-sensitive loss will be equivalent to the standard class cost-sensitive loss.

As for the values of the costs $C_{fp}$ and $C_{fn1},C_{fn2},\ldots ,C_{fnN}$, they are determined based on different specific tasks. For pedestrian detection, our intuition indicates that $C_{fn1},C_{fn2},\ldots ,C_{fnN}$ should be greater than $C_{fp}$, since miss detections are usually more difficult to be recovered than false positives, and $C_{fn1}$ should be greater than $C_{fn2}$, since lower-resolution samples are usually more difficult to be detected than higher-resolution ones, and so on for the case of $C_{fn(N-1)}$ and $C_{fnN}$. We will choose the optimal values of these costs experimentally via cross-validation. Then, when the values of $C_{fp}$ and $C_{fn1},C_{fn2},\ldots ,C_{fnN}$ are specified, we calculate the group cost-sensitive exponential loss as follows:

(11) $$\begin{array}{cc} E_{X,Y} & \left[I^{\prime }\left(y=1,x\in x_{G1}\right)\exp\left(-yC_{fn1}f\left(x\right)\right)\right.\\ & +I^{\prime }\left(y=1,x\in x_{G2}\right)\exp\left(-yC_{fn2}f\left(x\right)\right)\\ & +\cdots \\ & +I^{\prime }\left(y=1,x\in x_{GN}\right)\exp\left(-yC_{fnN}f\left(x\right)\right)\\ & \left.+I^{\prime }\left(y=-1,x\in x\right)\exp\left(-yC_{fp}f\left(x\right)\right)\right] \end{array}$$

where $I^{\prime }\left(\cdot \right)$ is an indicator function similar to Equation (7) but in an extended form:

(12) $$I^{\prime }\left(y=a,x\in b\right)=\left\{\begin{array}{cc} 1, & \mathrm{if}y=a\mathrm{and}x\in b\\ 0, & \mathrm{others} \end{array}\right.$$

#### 3.2.2. Group Cost-Sensitive Adaboost

Given the expected loss in Equation (11), the proposed group cost-sensitive AdaBoost algorithm is then derived by the gradient descent on its empirical estimate. Now we have a set of training samples ${\left\{(x_{i},y_{i})\right\}}_{i=1}^{n}$, the predictor f(x) as in Equation (3) and different resolution groups which are defined as follows:

(13) $$\begin{array}{cc} G_{+1}= & \left\{i|y_{i}=1,x_{i}\in x_{G1}\right\}\\ G_{+2}= & \left\{i|y_{i}=1,x_{i}\in x_{G2}\right\}\\ & \vdots \\ G_{+N}= & \left\{i|y_{i}=1,x_{i}\in x_{GN}\right\}\\ G_{-}= & \left\{i|y_{i}=-1\right\} \end{array}$$

At each iteration *m* in the boosting process, the selected weak learner consists of an optimal step $\alpha_{m}$ along the direction $g_{m}$ of the largest descent of the expected loss in Equation (11), and is expressed as:

(14) $$\begin{array}{cc} (\alpha_{m},g_{m})= & \arg\min_{\alpha ,g}\sum_{i\in G_{+1}}\omega_{i}^{\left(m\right)}\exp\left(-C_{fn1}\alpha g\left(x_{i}\right)\right)\\ & +\sum_{i\in G_{+2}}\omega_{i}^{\left(m\right)}\exp\left(-C_{fn2}\alpha g\left(x_{i}\right)\right)\\ & +\cdots +\sum_{i\in G_{+N}}\omega_{i}^{\left(m\right)}\exp\left(-C_{fnN}\alpha g\left(x_{i}\right)\right)\\ & +\sum_{i\in G_{-}}\omega_{i}^{\left(m\right)}\exp\left(C_{fp}\alpha g\left(x_{i}\right)\right) \end{array}$$

The optimal step α along the direction *g* is the solution of the following (Here we apply the gradient descent method to compute it; that is, we consider the output of the classifier for each training sample as a point ($f\left(x_{1}\right),\ldots ,f\left(x_{n}\right)$) in *n*-dimensional space, where each axis corresponds to a training sample, each weak learner g(x) corresponds to a vector of fixed orientation and length, and the goal is to reach the target point ($y_{1},\ldots ,y_{n}$) or any region where the value of the loss function is less than the value at that point in the least number of steps. Thus, we can perform the gradient descent optimization method to find g(x) with the steepest gradient and choose α to minimize test error, and this can be done efficiently with the standard scalar search procedures. See detailed proof in the Appendix A):

(15) $$\begin{array}{cc} & 2C_{fn1}\cdot err_{+1}\cdot \cosh\left(C_{fn1}\alpha \right)-C_{fn1}\cdot \Omega_{+1}\cdot e^{-C_{fn1}\alpha }\\ + & 2C_{fn2}\cdot err_{+2}\cdot \cosh\left(C_{fn2}\alpha \right)-C_{fn2}\cdot \Omega_{+2}\cdot e^{-C_{fn2}\alpha }\\ + & \cdots \\ + & 2C_{fnN}\cdot err_{+N}\cdot \cosh\left(C_{fnN}\alpha \right)-C_{fnN}\cdot \Omega_{+N}\cdot e^{-C_{fnN}\alpha }\\ + & 2C_{fp}\cdot err_{-}\cdot \cosh\left(C_{fp}\alpha \right)-C_{fp}\cdot \Omega_{-}\cdot e^{-C_{fp}\alpha }=0 \end{array}$$

with

(16) $$\begin{array}{cc} \Omega_{+1} & =\sum_{i\in G_{+1}}\omega_{i}^{\left(m\right)},\Omega_{+2}=\sum_{i\in G_{+2}}\omega_{i}^{\left(m\right)},\cdots ,\\ \Omega_{+N} & =\sum_{i\in G_{+N}}\omega_{i}^{\left(m\right)},\Omega_{-}=\sum_{i\in G_{-}}\omega_{i}^{\left(m\right)} \end{array}$$

(17) $$\begin{array}{cc} err_{+1}= & \sum_{i\in G_{+1}}\omega_{i}^{\left(m\right)}\left[1-I\left(y_{i}=g\left(x_{i}\right)\right)\right]\\ err_{+2}= & \sum_{i\in G_{+2}}\omega_{i}^{\left(m\right)}\left[1-I\left(y_{i}=g\left(x_{i}\right)\right)\right]\\ & \vdots \\ err_{+N}= & \sum_{i\in G_{+N}}\omega_{i}^{\left(m\right)}\left[1-I\left(y_{i}=g\left(x_{i}\right)\right)\right]\\ err_{-}= & \sum_{i\in G_{-}}\omega_{i}^{\left(m\right)}\left[1-I\left(y_{i}=g\left(x_{i}\right)\right)\right] \end{array}$$

After calculating the step α and the direction *g*, we can calculate the total loss of the weak learner (α,g) as follows:

(18) $$\begin{array}{cc} & err_{T}=\\ & \left(e^{C_{fn1}\alpha \left(g\right)}-e^{-C_{fn1}\alpha \left(g\right)}\right)\cdot err_{+1}+e^{-C_{fn1}\alpha \left(g\right)}\Omega_{+1}\\ + & \left(e^{C_{fn2}\alpha \left(g\right)}-e^{-C_{fn2}\alpha \left(g\right)}\right)\cdot err_{+2}+e^{-C_{fn2}\alpha \left(g\right)}\Omega_{+2}\\ + & \cdots \\ + & \left(e^{C_{fnN}\alpha \left(g\right)}-e^{-C_{fnN}\alpha \left(g\right)}\right)\cdot err_{+N}+e^{-C_{fnN}\alpha \left(g\right)}\Omega_{+N}\\ + & \left(e^{C_{fp}\alpha \left(g\right)}-e^{-C_{fp}\alpha \left(g\right)}\right)\cdot err_{-}+e^{-C_{fp}\alpha \left(g\right)}\Omega_{-} \end{array}$$

and select the direction of the largest descent so that the minimum loss is obtained:

(19) $$g_{m}=\arg\min_{g}\left(err_{T}\right)$$

Finally, we update the weight $\omega_{i}^{\left(m\right)}$ of each sample $x_{i}$ at the next iteration m+1 according to the following rules:

(20) $$\omega_{i}^{\left(m+1\right)}=\left\{\begin{array}{cc} \omega_{i}^{\left(m\right)}e^{-C_{fn1}\alpha_{m}g_{m}\left(x_{i}\right)}, & \mathrm{if}i\in G_{+1}\\ \omega_{i}^{\left(m\right)}e^{-C_{fn2}\alpha_{m}g_{m}\left(x_{i}\right)}, & \mathrm{if}i\in G_{+2}\\ \vdots & \vdots \\ \omega_{i}^{\left(m\right)}e^{-C_{fnN}\alpha_{m}g_{m}\left(x_{i}\right)}, & \mathrm{if}i\in G_{+N}\\ \omega_{i}^{\left(m\right)}e^{C_{fp}\alpha_{m}g_{m}\left(x_{i}\right)}, & \mathrm{if}i\in G_{-} \end{array}\right.$$

Briefly speaking, we define the possible descent directions using a set of weak learners ${\left\{g_{k}\left(x\right)\right\}}_{k=1}^{K}$, and obtain the optimal step α along each direction by solving Equation (15), which can be done efficiently with the standard scalar search procedures. The loss associated with the weak learner is then calculated as in Equation (18) when the step α and direction *g* are given, and the weak learner is selected in Equation (19) as the best one so that the minimum loss is achieved. We present in Algorithm 1 a summary of the proposed group cost-sensitive AdaBoost algorithm.

**Algorithm 1** Group Cost-Sensitive AdaBoost Algorithm**Input:** Training set ${\left\{(x_{i},y_{i})\right\}}_{i=1}^{n}$ where $x_{i}$ is the feature vector of the sample and $y_{i}\in \left\{1,-1\right\}$ is the class label, costs $\left\{C_{fn1},C_{fn2},\ldots ,C_{fnN},C_{fp}\right\}$ for different groups, the set of weak learners ${\left\{g_{k}\left(x\right)\right\}}_{k=1}^{K}$, and the number *M* of weak learners in the final classifier.**Output:** Strong classifier h(x) for multi-resolution detectors. 1: **Initialization:** Set of uniformly distributed weights for each group: 2: $\omega_{i}^{\left(0\right)}=\frac{1}{2|G_{+1}|},\forall i\in G_{+1};\;\omega_{i}^{\left(0\right)}=\frac{1}{2|G_{+2}|},\forall i\in G_{+2};\;\cdots ;\;\omega_{i}^{\left(0\right)}=\frac{1}{2|G_{+N}|},\forall i\in G_{+N};\;\omega_{i}^{\left(0\right)}=\frac{1}{2|G_{-}|},\forall i\in G_{-}$. 3: **for**
m={1,…,M}
**do** 4:     **for**
k={1,…,K}
**do** 5:         Compute parameter values as in Equations (16), (17) with $g\left(x\right)=g_{k}\left(x\right)$; 6:         Obtain the value of $\alpha_{k}$ by solving Equation (15); 7:         Calculate the loss of the weak learner $\left(\alpha_{k},g_{k}\left(x\right)\right)$ as in Equation (18). 8:     **end for** 9:     Select the best weak learner $\left(\alpha_{m},g_{m}\left(x\right)\right)$ with the minimum loss as in Equation (19);10:     Update the weights $\omega_{i}$ according to Equation (20).11: **end for**12: **return**$h\left(x\right)=\mathrm{sgn}\left[\sum_{m=1}^{M}\alpha_{m}g_{m}\left(x\right)\right]$.

### 3.3. Multi-Resolution Detectors

By integrating the proposed group cost-sensitive AdaBoost algorithm into the baseline LDCF and CCF frameworks, respectively, i.e., replacing the standard AdaBoost algorithm used in LDCF and CCF by the proposed group cost-sensitive AdaBoost algorithm, a new group cost-sensitive LDCF detector and a new group cost-sensitive CCF detector (denoted as “GCS-LDCF” and “GCS-CCF” in the following experiments, respectively) can be obtained to better handle the detection in multi-resolution conditions. To perform multi-resolution pedestrian detection, we applied the proposed detectors on each test image with a multi-scale sliding window strategy, and adopted non-maximal suppression to merge multiple nearby detections to obtain the final detection results.

## 4. Experimental Evaluation

To evaluate the proposed approaches, we conducted the experiments on two standard datasets: the Caltech pedestrian detection benchmark [2], and the KAIST multispectral pedestrian detection benchmark [16].

The Caltech benchmark is by far the largest and the most challenging pedestrian dataset, by taking a video around 10 h long (640×480, 30 Hz) from a vehicle driving through regular traffic in an urban environment. This dataset contains a large number of pedestrians, i.e., a total number of 350,000 annotated bounding boxes and 2300 unique pedestrians. However, it is challenging due to realistic occlusion frequency and many low-resolution pedestrians.

The KAIST benchmark is a multispectral pedestrian dataset. Different from the Caltech benchmark that contains only color images, this benchmark captures the additional thermal images and consists of 95 k color-thermal pairs (640×480, 20 Hz) taken from a vehicle. All the pairs are manually annotated (person, people, cyclist) for the total of 103,128 dense annotations and 1182 unique pedestrians. The annotation includes temporal correspondence between bounding boxes which are similar to the Caltech benchmark.

### 4.1. Experimental Setup

The common experimental setups are followed on each of two benchmarks: For Caltech, its training set (set00–set05) is used to train the detectors, and its test set (set06–set10) is used to obtain the detection results; for KAIST, its training set (set00–set05) is used to train the detectors and the detection results are reported on its test set (set06–set11). For detector training, we chose the image regions labeled as “persons” that were non-occluded with different resolutions as positive samples, and chose the patches of random sizes at random locations in the training images without pedestrians as negative samples.

The important parameters of the proposed approach during training were set as follows: we considered two resolution groups (N=2)—low-resolution samples (30–80 pixels high in Caltech, 30–115 pixels high in KAIST) and high-resolution samples (taller than 80 pixels in Caltech, taller than 115 pixels in KAIST), as defined in each of two benchmarks. As for the optimal value of the costs for different resolution groups, they were selected from $C_{fp}=1$, $C_{fn2}\in \left[C_{fp}:0.1:10\right]$ and $C_{fn1}\in \left[C_{fn2}:0.1:C_{fn2}+10\right]$ experimentally by cross-validation. To construct a strong classifier, 4096 weak classifiers were trained and combined via the proposed boosting algorithm, and a pool of random candidate regions from image samples were used to construct the nodes of these decision trees. The multi-scale models were used to increase scale invariance, and three bootstrapping stages were applied with 25,000 additional hard negative samples each time.

To evaluate the results, we used the ground truth annotations and evaluation code available on the website of the Caltech benchmark (www.vision.caltech.edu/Image_Datasets/CaltechPedestrians/) and the KAIST benchmark (https://sites.google.com/site/pedestrianbenchmark/), respectively. For both benchmarks, the same per-image evaluation methodology was adopted—that is, the miss rate vs. FPPI (False-Positive-Per-Image) curves were used to compare the results. In addition, to compare different approaches more conveniently, we also calculated their summarized performances in terms of the *log-average miss rate*, which is the average of the miss rates at several fixed FPPI points (The mean miss rate at 0.0100, 0.0178, 0.0316, 0.0562, 0.1000, 0.1778, 0.3162, 0.5623 and 1.0000 FPPI). evenly distributed in the log-space from $10^{-2}$ to $10^{0}$. Different test subsets are available on two benchmarks to evaluate detectors in different conditions. In order to validate the effectiveness of the proposed approach for multi-resolution detection, we mainly conducted the experiments on several resolution-specific subsets: for Caltech, including the popular “Reasonable” (pedestrians of ≥50 pixels high and less than 35% occluded), “Large-Scale” (pedestrians of ≥100 pixels high and non-occluded), “Near-Scale” (pedestrians of ≥80 pixels high and non-occluded), and “Medium-Scale” (pedestrians of 30–80 pixels high and non-occluded); for KAIST, including the popular “Reasonable All” (pedestrians of ≥55 pixels high and less than 50% occluded), “Near-Scale” (pedestrians of ≥115 pixels high and non-occluded), “Medium-Scale” (pedestrians of 45–115 pixels high and non-occluded), and “Far-Scale” (pedestrians of ≤45 pixels high and non-occluded).

### 4.2. Comparison with Popular Approaches on the Caltech Benchmark

The proposed approaches are compared on the Caltech benchmark with many popular pedestrian detection approaches in the literature, including (the detailed definitions of the following short forms can be found in www.vision.caltech.edu/Image_Datasets/CaltechPedestrians/) VJ [17], HOG [18], ChnFtrs [19], ConvNet [20], FPDW [21], LatSVM [22], pAUCBoost [23], RandForest [24], SDN [25], DBN-Mut [26], Franken [27], JointDeep [28], InformedHaar [29], LDCF [6], ACF-Caltech+ [6], SpatialPooling [30], SpatialPooling+ [31], Katamari [32], LFOV [33], NAMC [34], DeepCascade [35], SCCPriors [36], Checkerboards [37], DeepParts [38], and CompACT-Deep [39]. The results of these approaches were obtained directly from the same website as the evaluation code. Note that some recent methods, such as AdaptFasterRCNN [40], SA-FastRCNN [41], F-DNN2 [42], TLL-TFA [43], and SDS-RCNN [44] were not considered in comparisons since they require additional external data (e.g., ImageNet, CityPersons, Cityscapes, TudBrussels, ETH) to train their deep models.

**Figure 3:**
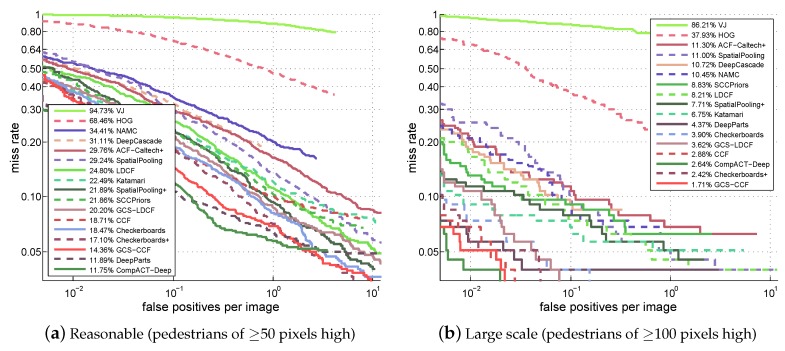

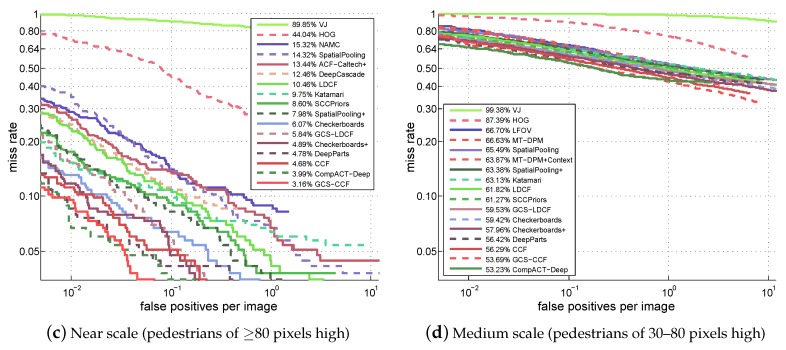
Comparison with popular approaches on the Caltech benchmark.

For the results, the miss rate vs. FPPI curves and their corresponding *log-average miss rates* (reported in the figure legend) of different approaches on four test sets of the Caltech benchmark are shown in Figure 3. Due to the space limitation, only the results of top 15 approaches plus the classic VJ and HOG are presented in the figure. It can be clearly seen that: (1) The proposed GCS-LDCF obviously performs better than its baseline LDCF on four test sets, i.e., 4.60 percentage points better on the “Reasonable” set, 4.59 percentage points better on the “Large-Scale” set, 4.62 percentage points better on the “Near-Scale” set, and 2.29 percentage points better on the “Medium-Scale” set, respectively. (2) The proposed GCS-CCF also clearly outperforms its baseline CCF on four test sets, i.e., 4.35 percentage points better on the “Reasonable” set, 1.17 percentage points better on the “Large-Scale” set, 1.52 percentage points better on the “Near-Scale” set, and 2.60 percentage points better on the “Medium-Scale” set, respectively. (3) These are positive demonstrations that the proposed approaches truly benefit from exploring different costs for the sample groups with different resolutions by the group cost-sensitive AdaBoost algorithm in the training process; (4) according to the miss rate vs. FPPI curves and the *log-average miss rates* on four test sets, the proposed GCS-CCF approach outperforms most other popular approaches, validating that it is an effective method for pedestrian detection, especially in multi-resolution occasions; (5) note that some well-performing approaches utilize additional motion or context information or multiple-feature combinations to aid detection (e.g., the CompACT-Deep approach [39] combines the ACF, SS, CB, LDA, and CNN features to learn cascades; the Checkerboards+ approach [37] uses the flow-based motion features from [45]), while the proposed approach in this paper focuses on pedestrian detection in static images and does not take such kinds of information into consideration. Nevertheless, utilizing motion and context information or additional features in the proposed approach for further improvement is a potential area for future research.

### 4.3. Comparison with Popular Approaches on the KAIST Benchmark

The proposed approaches were also compared on the KAIST benchmark with some popular pedestrian detection approaches. Since the KAIST benchmark is a recently released pedestrian dataset, the results in the literature are not as many as the Caltech benchmark. Thus, we mainly made comparisons with several baseline approaches proposed in [16]. Also note that, different from the Caltech benchmark, the KAIST benchmark is a multispectral pedestrian dataset (color channels + thermal channel); thus, the baseline approaches extend the popular ACF framework [46] to handle both color and additional thermal channels. To make fair comparisons, we therefore also extend the proposed GCS-LDCF and GCS-CCF approaches to “GCS-LDCF+T+THOG” and “GCS-CCF+T+THOG”, respectively, with additional thermal channels by following the same method as explained in Section 3.2 in [16].

**Figure 4:**
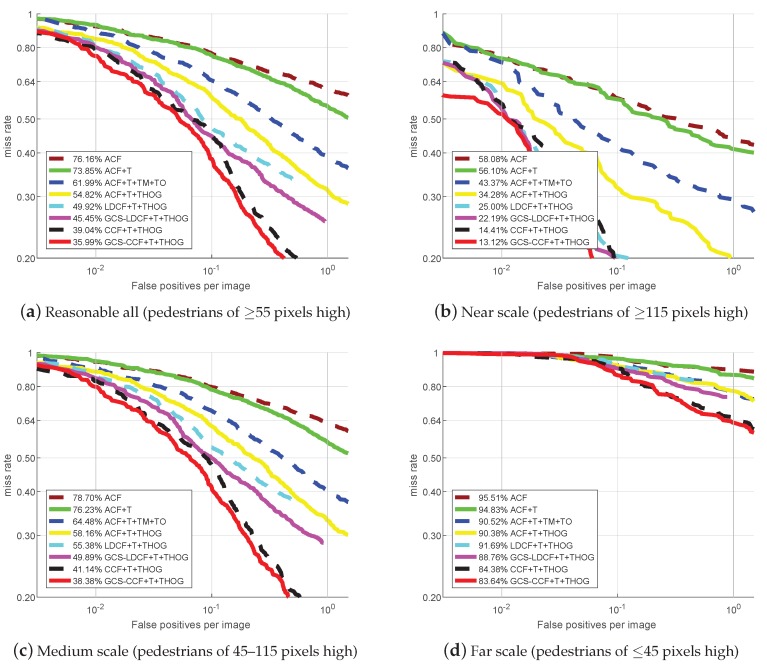
Comparison with popular approaches on the KAIST benchmark.

Figure 4 presents the miss rate vs. FPPI curves and their corresponding *log-average miss rates* (reported in the figure legend) of different approaches on four test sets of the KAIST benchmark. We can observe that: (1) The best-performing approach in [16] is ACF+T+THOG. By replacing the ACF detector with the LDCF detector and the CCF detector, our baseline LDCF+T+THOG and CCF+T+THOG already outperforms the ACF+T+THOG approach on four test sets. (2) The proposed GCS-LDCF+T+THOG approach also performs better than the baseline LDCF+T+THOG on four test sets (4.47 percentage points better on “Reasonable All”, 2.81 percentage points better on the “Near Scale”, 5.49 percentage points better on the “Medium-Scale”, and 2.93 percentage points better on the “Far Scale”, respectively). (3) The proposed GCS-CCF+T+THOG approach further outperforms the baseline CCF+T+THOG on four test sets (3.05 percentage points better on “Reasonable All”, 1.29 percentage points better on the “Near Scale”, 2.76 percentage points better on the “Medium Scale”, and 0.74 percentage points better on the “Far Scale”, respectively). (4) These results validate the effectiveness of the proposed group cost-sensitive boosting algorithm, and show that it also provides an effective way for multi-resolution pedestrian detection in multispectral conditions.

### 4.4. Discussion: Influence of Group Number

The number of resolution groups is an important factor in the proposed approaches, and there is no prior knowledge about the optimal number of groups. Therefore, we empirically selected the best number of groups on the Caltech and KAIST benchmarks, respectively, by changing the group number (*N*) from 0 to 4 in the proposed GCS-LDCF and GCS-CCF approaches, and comparing their performances. N=0 means that we do not distinguish false negatives from false positives in wrong detections, which equals to the original LDCF and CCF approaches. N=1 means that we consider all positive samples as a group and assign different costs for false negatives and false positives, respectively, which equals to the standard cost-sensitive setting. N>1 means that we further divide positive samples into different resolution groups and assign different costs for them. Specifically, when N=2, the positive samples are divided into group 1 (30–80 pixels high in Caltech, 30–115 pixels high in KAIST) and group 2 (taller than 80 pixels in Caltech, taller than 115 pixels in KAIST); when N=3, the positive samples are divided into group 1 (30–50 pixels high in Caltech, 30–55 pixels high in KAIST), group 2 (50–80 pixels high in Caltech, 55–115 pixels high in KAIST), and group 3 (taller than 80 pixels in Caltech, taller than 115 pixels in KAIST); and when N=4, the positive samples are divided into group 1 (less than 30 pixels high in Caltech, less than 30 pixels high in KAIST), group 2 (30–50 pixels high in Caltech, 30–55 pixels high in KAIST), group 3 (50–80 pixels high in Caltech, 55–115 pixels high in KAIST), and group 4 (taller than 80 pixels in Caltech, taller than 115 pixels in KAIST).

**Figure 5:**
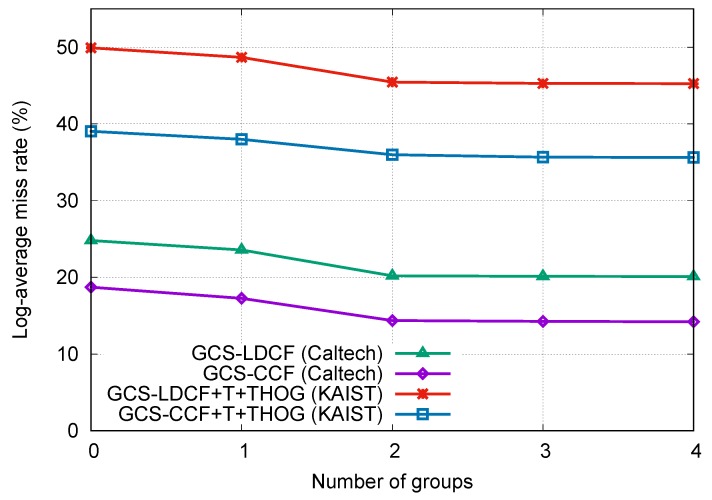
Influence of group number in GCS-LDCF and GCS-CCF on the Caltech and KAIST benchmarks, respectively.

The results are shown in Figure 5. We can observe that: (1) The performances with one group are better than the performances with no group, indicating the positive effect of distinguishing false negatives from false positives in wrong detections when training. (2) When the group number is increased from 1 to 2, the performances are also clearly improved, validating the effectiveness of the proposed group cost-sensitive boosting algorithm. (3) The performance gains become slight when the group number continues to increase from 2 to 3, and shows no improvement when the group number changes from 3 to 4. We think the main reasons for this may lie in that when we increase the group number, more positive samples with low resolution are divided and considered; however, according to [2], pedestrians less than 50 pixels high are very difficult to recognize, and for pedestrians below 30 pixels, even human annotators have difficulty in identifying them reliably. Moreover, the number of pedestrian samples below 30 pixels in both datasets is small. Therefore, very low-resolution samples (less than 50 pixels high) in Caltech and KAIST are in the minority and naturally difficult to detect, and thus can hardly provide help in the proposed approaches, which is why we chose group number N=2 in previous experiments. Overall, we can learn that the optimal number of resolution groups could depend on the specific detection tasks, as well as the data distribution of the specific datasets.

### 4.5. Discussion: Performance on Very Low-Resolution Samples

There are reasons why not only the proposed approaches, but also all the other ones perform poorly on very low-resolution pedestrian samples, as shown in Figure 3d and Figure 4d. According to the authors’ claim in [2], pedestrians less than 50 pixels tall in the Caltech benchmark are very difficult to recognize due to the missing appearance details, and for the pedestrians around 30 pixels, even human annotators have difficulty in identifying them reliably. This is also the case in the KAIST benchmark, since it is constructed in a similar way to Caltech. That is why there are “Reasonable” settings in both benchmarks (pedestrians taller than 50 pixels in Caltech and taller than 55 pixels in KAIST), because the pedestrians less than 50 pixels tall are naturally very difficult to detect. Therefore, the detection performances of all the approaches for these samples are far from satisfactory. This also can explain why the performances in Figure 4d are even poorer (>80% mean miss rate) than those in Figure 3d (>50% mean miss rate), since the samples in Figure 4d contain only the pedestrians less than 45 pixels tall, which are naturally very difficult to detect, but the samples in Figure 3d contain the pedestrians between 30 and 80 pixels tall, where the parts which are 30–50 tall are difficult to detect, while the parts which are 50–80 tall are relatively easier to detect.

As for our proposed approaches, according to the results in Figure 3d, GCS-LDCF and GCS-CCF still outperform the baseline LDCF and CCF (2.29 and 2.60 percentage points, respectively, which are relatively clear improvements considering the small performance gap between different approaches), and we believe the benefits come from the pedestrian samples of 50–80 pixels tall which provide actual help in the proposed group cost-sensitive boosting algorithm. However, due to other pedestrian samples that are 30–50 pixels tall which are difficult to detect and thus can hardly provide help in the proposed algorithm, the overall performances of GCS-LDCF and GCS-CCF are still not good enough in this case.

Overall, based on the experimental results, we can say that the proposed approaches could truly provide performance gain on low-resolution pedestrian samples (50–80 pixels tall). But for very low-resolution pedestrian samples (less than 50 pixels tall), since their detection is naturally a hard problem, there are still no good solutions for solving it, and the proposed approaches are clearly not the best solutions, but at least provide an effort to address this problem.

### 4.6. Runtime Analysis

**Figure 6:**
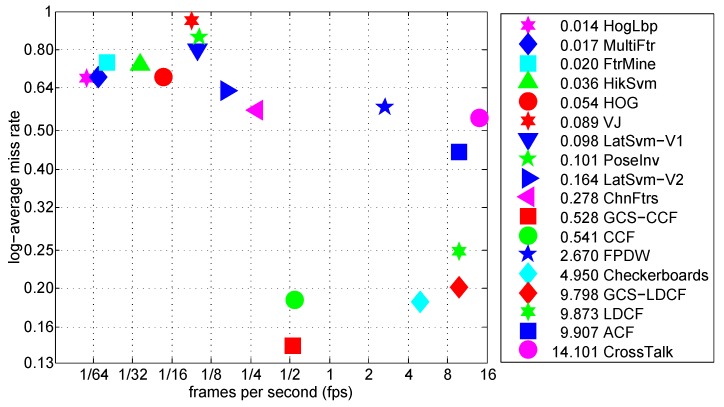
Log-average miss rate vs. runtime of different approaches on Caltech “Reasonable” setting (symbols closer to the bottom-right corner indicating that the corresponding approaches possess both better accuracy and faster runtime speed).

In this section, we compare the runtime of the proposed approaches with other methods in the literature using video frames from the Caltech benchmark. The frames had a resolution of 640×480 pixels, and the runtime was measured by averaging the runtime over multiple frames with the “Reasonable” settings. The runtimes of other approaches were obtained from [2], where runtimes of all detectors were normalized to the rate of a single modern machine, so that all times were directly comparable. In Figure 6, we plot *log-average miss rate* versus runtime for each approach. Note that symbols closer to the bottom-right corner indicate that the corresponding approaches possess both better accuracy and faster runtime speed. We can see that the proposed GCS-LDCF approach runs faster than most other detectors, and runs as fast as the original LDCF approach but improves its accuracy by almost 5 percentage points. As for the proposed GCS-CCF approach, its runtime speed is almost the same as the original CCF approach. Due to the sliding-window mechanism and deep-learning-based feature computation in a huge number of windows, their runtime speed is now around 0.5 fps. However, considering their good detection accuracy compared to other approaches, and the fact that acceleration techniques used in Fast R-CNN [47] are also applicable to CCF and GCS-CCF, it is very valuable and has the possibility of further improving their runtime speed. This will be done in our work in the future.

## 5. Conclusions

In this paper, we proposed a new group cost-sensitive boosting algorithm for handling multi-resolution pedestrian detection. Different from the traditional boosting-based approaches where low-resolution samples are treated with equal importance as high-resolution ones, thus resulting in false-negatives since they are more easily rejected in the early stages during boosting, the proposed approach extends the standard AdaBoost algorithm by further exploring different costs for different resolution groups of the samples in the boosting process, and placing greater emphasis on low-resolution samples, which are usually more difficult to be detected, in order to better handle the detection in multi-resolution conditions. The effectiveness of the proposed approach has been validated by its promising performance compared to other popular methods on different resolution-specific test sets of the Caltech pedestrian benchmark and the KAIST multispectral pedestrian benchmark.

Future work includes the extension of the proposed group cost-sensitive boosting algorithm to the application of general object detection, and the utilization of additional motion and context information or other powerful features in the proposed approach for further performance improvement, as well as acceleration of the GCS-CCF approach while keeping its high detection accuracy.

## Figures and Tables

**Table 1:** A list of the terms that appear in our approach.

Term	Definition	Meaning in Pedestrian Detection Problem
*x*	Feature representation of a sample	Image region that needs to be classified as pedestrian or not
*y*	Class label of a sample	Its value will be 1 if the corresponding image region *x* is pedestrian, otherwise 0
h(x)	Detector (binary classifier)	Get label y given image region *x*
f(x)	Predictor (strong classifier learned via boosting)	Output score given image region *x* (*x* will be pedestrian if score is positive, otherwise non-pedestrian)
Loss	Loss function	A measurement for wrong detections (pedestrian region is classified as non-pedestrian or background region is classified as pedestrian)
g(x)	Weak classifier in boosting learning	Simple classifier to decide if an image region is pedestrian (only slightly better than random)
α	Weight of each weak classifier	
ω	Weight of each sample	Its value will be increased if detection is wrong, otherwise decreased
*C*	Costs in group cost-sensitive loss function	Different cost values are assigned to measure wrong detections in different resolution pedestrian samples
G	Groups of different resolution samples	Image regions are divided into groups according to different resolution pedestrians in it
Ω	Sum of weights of samples in each resolution group	
err	Classification error	Total loss of detections in each resolution pedestrian group

## Appendix A. Proof of the Conclusion in Equation (15)

Given the expected loss as in Equation (11), the indicator function as in Equation (7) and the extended indicator function as in Equation (12), the group-sensitive cost function can be expressed as:

$$\begin{array}{cc} J[f]= & E_{X,Y}\left[I^{\prime }\left(y=1,x\in x_{G1}\right)e^{-C_{fn1}f\left(x\right)}\right.\\ & +I^{\prime }\left(y=1,x\in x_{G2}\right)e^{-C_{fn2}f\left(x\right)}\\ & +\cdots \\ & +I^{\prime }\left(y=1,x\in x_{GN}\right)e^{-C_{fnN}f\left(x\right)}\\ & \left.+I^{\prime }\left(y=-1,x\in x\right)e^{C_{fp}f\left(x\right)}\right] \end{array}$$

and by adding the weak learner αg(x) to the predictor f(x), we have:

$$\begin{array}{cc} J & [f+\alpha g]=\\ & E_{X,Y}\left[I^{\prime }\left(y=1,x\in x_{G1}\right)e^{-C_{fn1}f\left(x\right)}e^{-C_{fn1}\alpha g\left(x\right)}\right.\\ & +I^{\prime }\left(y=1,x\in x_{G2}\right)e^{-C_{fn2}f\left(x\right)}e^{-C_{fn2}\alpha g\left(x\right)}\\ & +\cdots \\ & +I^{\prime }\left(y=1,x\in x_{GN}\right)e^{-C_{fnN}f\left(x\right)}e^{-C_{fnN}\alpha g\left(x\right)}\\ & \left.+I^{\prime }\left(y=-1,x\in x\right)e^{C_{fp}f\left(x\right)}e^{C_{fp}\alpha g\left(x\right)}\right] \end{array}$$

Since J[f+αg] is minimized if and only if the argument of the expectation is minimized for all x, the direction of the largest descent and optimal step size can be obtained by:

$$\begin{array}{cc} (\alpha_{m}, & g_{m}\left(x\right))=\arg\min_{\alpha ,g(x)}\{\\ & E_{Y|X}\left[I^{\prime }\left(y=1,x\in x_{G1}\right)e^{-C_{fn1}f\left(x\right)}e^{-C_{fn1}\alpha g\left(x\right)}\right.\\ & +I^{\prime }\left(y=1,x\in x_{G2}\right)e^{-C_{fn2}f\left(x\right)}e^{-C_{fn2}\alpha g\left(x\right)}\\ & +\cdots \\ & +I^{\prime }\left(y=1,x\in x_{GN}\right)e^{-C_{fnN}f\left(x\right)}e^{-C_{fnN}\alpha g\left(x\right)}\\ & +\left.\left.I^{\prime }\left(y=-1,x\in x\right)e^{C_{fp}f\left(x\right)}e^{C_{fp}\alpha g\left(x\right)}|x\right]\right\} \end{array}$$

The expectation can be expressed as follows:

$$\begin{array}{cc} & E_{Y|X}\left[I^{\prime }\left(y=1,x\in x_{G1}\right)e^{-C_{fn1}f\left(x\right)}e^{-C_{fn1}\alpha g\left(x\right)}\right.\\ + & I^{\prime }\left(y=1,x\in x_{G2}\right)e^{-C_{fn2}f\left(x\right)}e^{-C_{fn2}\alpha g\left(x\right)}\\ + & \cdots \\ + & I^{\prime }\left(y=1,x\in x_{GN}\right)e^{-C_{fnN}f\left(x\right)}e^{-C_{fnN}\alpha g\left(x\right)}\\ + & \left.I^{\prime }\left(y=-1,x\in x\right)e^{C_{fp}f\left(x\right)}e^{C_{fp}\alpha g\left(x\right)}|x\right] \end{array}$$

$$\begin{array}{cc} = & E_{Y|X}\left[I^{\prime }\left(y=1,x\in x_{G1}\right)I\left(g\left(x_{G1}\right)=1\right)e^{-C_{fn1}f\left(x\right)}e^{-C_{fn1}\alpha }\right.\\ + & I^{\prime }\left(y=1,x\in x_{G1}\right)I\left(g\left(x_{G1}\right)=-1\right)e^{-C_{fn1}f\left(x\right)}e^{C_{fn1}\alpha }\\ + & I^{\prime }\left(y=1,x\in x_{G2}\right)I\left(g\left(x_{G2}\right)=1\right)e^{-C_{fn2}f\left(x\right)}e^{-C_{fn2}\alpha }\\ + & I^{\prime }\left(y=1,x\in x_{G2}\right)I\left(g\left(x_{G2}\right)=-1\right)e^{-C_{fn2}f\left(x\right)}e^{C_{fn2}\alpha }\\ + & \cdots \\ + & I^{\prime }\left(y=1,x\in x_{GN}\right)I\left(g\left(x_{GN}\right)=1\right)e^{-C_{fnN}f\left(x\right)}e^{-C_{fnN}\alpha }\\ + & I^{\prime }\left(y=1,x\in x_{GN}\right)I\left(g\left(x_{GN}\right)=-1\right)e^{-C_{fnN}f\left(x\right)}e^{C_{fnN}\alpha }\\ + & I^{\prime }\left(y=-1,x\in x\right)I\left(g\left(x\right)=1\right)e^{C_{fp}f\left(x\right)}e^{C_{fp}\alpha }\\ + & \left.I^{\prime }\left(y=-1,x\in x\right)I\left(g\left(x\right)=-1\right)e^{C_{fp}f\left(x\right)}e^{-C_{fp}\alpha }|x\right] \end{array}$$

$$\begin{array}{cc} = & E_{Y|X}\\ & \left[I^{\prime }\left(y=1,x\in x_{G1}\right)I\left(g\left(x_{G1}\right)=-1\right)e^{-C_{fn1}f\left(x\right)}\left(e^{C_{fn1}\alpha }-e^{-C_{fn1}\alpha }\right)\right.\\ + & I^{\prime }\left(y=1,x\in x_{G1}\right)e^{-C_{fn1}f\left(x\right)}e^{-C_{fn1}\alpha }\\ + & I^{\prime }\left(y=1,x\in x_{G2}\right)I\left(g\left(x_{G2}\right)=-1\right)e^{-C_{fn2}f\left(x\right)}\left(e^{C_{fn2}\alpha }-e^{-C_{fn2}\alpha }\right)\\ + & I^{\prime }\left(y=1,x\in x_{G2}\right)e^{-C_{fn2}f\left(x\right)}e^{-C_{fn2}\alpha }\\ + & \cdots \\ + & I^{\prime }\left(y=1,x\in x_{GN}\right)I\left(g\left(x_{GN}\right)=-1\right)e^{-C_{fnN}f\left(x\right)}\left(e^{C_{fnN}\alpha }-e^{-C_{fnN}\alpha }\right)\\ + & I^{\prime }\left(y=1,x\in x_{GN}\right)e^{-C_{fnN}f\left(x\right)}e^{-C_{fnN}\alpha }\\ + & I^{\prime }\left(y=-1,x\in x\right)I\left(g\left(x\right)=1\right)e^{C_{fp}f\left(x\right)}\left(e^{C_{fp}\alpha }-e^{-C_{fp}\alpha }\right)\\ + & \left.I^{\prime }\left(y=-1,x\in x\right)e^{C_{fp}f\left(x\right)}e^{-C_{fp}\alpha }|x\right] \end{array}$$

$$\begin{array}{cc} = & P_{Y|X}\left(1|x_{G1}\right)e^{-C_{fn1}f\left(x\right)}I\left(g\left(x_{G1}\right)=-1\right)\left(e^{C_{fn1}\alpha }-e^{-C_{fn1}\alpha }\right)\\ + & P_{Y|X}\left(1|x_{G1}\right)e^{-C_{fn1}f\left(x\right)}e^{-C_{fn1}\alpha }\\ + & P_{Y|X}\left(1|x_{G2}\right)e^{-C_{fn2}f\left(x\right)}I\left(g\left(x_{G2}\right)=-1\right)\left(e^{C_{fn2}\alpha }-e^{-C_{fn2}\alpha }\right)\\ + & P_{Y|X}\left(1|x_{G2}\right)e^{-C_{fn2}f\left(x\right)}e^{-C_{fn2}\alpha }\\ + & \cdots \\ + & P_{Y|X}\left(1|x_{GN}\right)e^{-C_{fnN}f\left(x\right)}I\left(g\left(x_{GN}\right)=-1\right)\left(e^{C_{fnN}\alpha }-e^{-C_{fnN}\alpha }\right)\\ + & P_{Y|X}\left(1|x_{GN}\right)e^{-C_{fnN}f\left(x\right)}e^{-C_{fnN}\alpha }\\ + & P_{Y|X}\left(-1|x\right)e^{C_{fp}f\left(x\right)}I\left(g\left(x\right)=1\right)\left(e^{C_{fp}\alpha }-e^{-C_{fp}\alpha }\right)\\ + & P_{Y|X}\left(-1|x\right)e^{C_{fp}f\left(x\right)}e^{-C_{fp}\alpha } \end{array}$$

thus the direction of the largest descent and optimal step size can be obtained by:

$$\begin{array}{cc} (\alpha_{m}, & g_{m}\left(x\right))=\arg\min_{\alpha ,g(x)}\\ & P_{Y|X}^{\prime }\left(1|x_{G1}\right)I\left(g\left(x_{G1}\right)=-1\right)\left(e^{C_{fn1}\alpha }-e^{-C_{fn1}\alpha }\right)\\ & +P_{Y|X}^{\prime }\left(1|x_{G1}\right)e^{-C_{fn1}\alpha }\\ & +P_{Y|X}^{\prime }\left(1|x_{G2}\right)I\left(g\left(x_{G2}\right)=-1\right)\left(e^{C_{fn2}\alpha }-e^{-C_{fn2}\alpha }\right)\\ & +P_{Y|X}^{\prime }\left(1|x_{G2}\right)e^{-C_{fn2}\alpha }\\ & +\cdots \\ & +P_{Y|X}^{\prime }\left(1|x_{GN}\right)I\left(g\left(x_{GN}\right)=-1\right)\left(e^{C_{fnN}\alpha }-e^{-C_{fnN}\alpha }\right)\\ & +P_{Y|X}^{\prime }\left(1|x_{GN}\right)e^{-C_{fnN}\alpha }\\ & +P_{Y|X}^{\prime }\left(-1|x\right)I\left(g\left(x\right)=1\right)\left(e^{C_{fp}\alpha }-e^{-C_{fp}\alpha }\right)\\ & \left.+P_{Y|X}^{\prime }\left(-1|x\right)e^{-C_{fp}\alpha }\right\} \end{array}$$

where

$$\begin{array}{cc} P_{Y|X}^{\prime }\left(1|x_{G1}\right)= & P_{Y|X}\left(1|x_{G1}\right)e^{-C_{fn1}f\left(x\right)}/P_{sum}\\ P_{Y|X}^{\prime }\left(1|x_{G2}\right)= & P_{Y|X}\left(1|x_{G2}\right)e^{-C_{fn2}f\left(x\right)}/P_{sum}\\ & \vdots \\ P_{Y|X}^{\prime }\left(1|x_{GN}\right)= & P_{Y|X}\left(1|x_{GN}\right)e^{-C_{fnN}f\left(x\right)}/P_{sum}\\ P_{Y|X}^{\prime }\left(-1|x\right)= & P_{Y|X}\left(-1|x\right)e^{C_{fp}f\left(x\right)}/P_{sum} \end{array}$$

with

$$\begin{array}{cc} P_{sum}= & P_{Y|X}\left(1|x_{G1}\right)e^{-C_{fn1}f\left(x\right)}+P_{Y|X}\left(1|x_{G2}\right)e^{-C_{fn2}f\left(x\right)}\\ & +\cdots +P_{Y|X}\left(1|x_{GN}\right)e^{-C_{fnN}f\left(x\right)}\\ & +P_{Y|X}\left(-1|x\right)e^{C_{fp}f\left(x\right)} \end{array}$$

are the posterior estimates associated with each sample. Hence, the weak learner with the minimum cost can be obtained by:

$$\begin{array}{cc} (\alpha_{m}, & g_{m}\left(x\right))=\arg\min_{\alpha ,g(x)}\\ & E_{X}\left\{P_{Y|X}^{\prime }\left(1|x_{G1}\right)I\left(g\left(x_{G1}\right)=-1\right)\left(e^{C_{fn1}\alpha }-e^{-C_{fn1}\alpha }\right)\right.\\ & +P_{Y|X}^{\prime }\left(1|x_{G1}\right)e^{-C_{fn1}\alpha }\\ & +P_{Y|X}^{\prime }\left(1|x_{G2}\right)I\left(g\left(x_{G2}\right)=-1\right)\left(e^{C_{fn2}\alpha }-e^{-C_{fn2}\alpha }\right)\\ & +P_{Y|X}^{\prime }\left(1|x_{G2}\right)e^{-C_{fn2}\alpha }\\ & +\cdots \\ & +P_{Y|X}^{\prime }\left(1|x_{GN}\right)I\left(g\left(x_{GN}\right)=-1\right)\left(e^{C_{fnN}\alpha }-e^{-C_{fnN}\alpha }\right)\\ & +P_{Y|X}^{\prime }\left(1|x_{GN}\right)e^{-C_{fnN}\alpha }\\ & +P_{Y|X}^{\prime }\left(-1|x\right)I\left(g\left(x\right)=1\right)\left(e^{C_{fp}\alpha }-e^{-C_{fp}\alpha }\right)\\ & \left.+P_{Y|X}^{\prime }\left(-1|x\right)e^{-C_{fp}\alpha }\right\} \end{array}$$

Given the definitions in Equations (16) and (17), and by replacing the expectations with the sample averages, we have:

$$\begin{array}{cc} (\alpha_{m}, & g_{m})=\arg\min_{\alpha ,g}\\ & \left\{\left(e^{C_{fn1}\alpha }-e^{-C_{fn1}\alpha }\right)\cdot err_{+1}+e^{-C_{fn1}\alpha }\Omega_{+1}\right.\\ & +\left(e^{C_{fn2}\alpha }-e^{-C_{fn2}\alpha }\right)\cdot err_{+2}+e^{-C_{fn2}\alpha }\Omega_{+2}\\ & +\cdots \\ & +\left(e^{C_{fnN}\alpha }-e^{-C_{fnN}\alpha }\right)\cdot err_{+N}+e^{-C_{fnN}\alpha }\Omega_{+N}\\ & +\left.\left(e^{C_{fp}\alpha }-e^{-C_{fp}\alpha }\right)\cdot err_{-}+e^{-C_{fp}\alpha }\Omega_{-}\right\} \end{array}$$

Given the direction of the largest descent g(x), and by setting the derivative with respect to α to zero:

$$\begin{array}{cc} \frac{\partial }{\partial \alpha } & =C_{fn1}\left(e^{C_{fn1}\alpha }+e^{-C_{fn1}\alpha }\right)\cdot err_{+1}-C_{fn1}e^{-C_{fn1}\alpha }\Omega_{+1}\\ + & C_{fn2}\left(e^{C_{fn2}\alpha }+e^{-C_{fn2}\alpha }\right)\cdot err_{+2}-C_{fn2}e^{-C_{fn2}\alpha }\Omega_{+2}\\ + & \cdots \\ + & C_{fnN}\left(e^{C_{fnN}\alpha }+e^{-C_{fnN}\alpha }\right)\cdot err_{+N}-C_{fnN}e^{-C_{fnN}\alpha }\Omega_{+N}\\ + & C_{fp}\left(e^{C_{fp}\alpha }+e^{-C_{fp}\alpha }\right)\cdot err_{-}-C_{fp}e^{-C_{fp}\alpha }\Omega_{-}=0 \end{array}$$

thus the optimal step size α is the solution of:

$$\begin{array}{cc} & 2C_{fn1}\cdot err_{+1}\cdot \cosh\left(C_{fn1}\alpha \right)-C_{fn1}\cdot \Omega_{+1}\cdot e^{-C_{fn1}\alpha }\\ + & 2C_{fn2}\cdot err_{+2}\cdot \cosh\left(C_{fn2}\alpha \right)-C_{fn2}\cdot \Omega_{+2}\cdot e^{-C_{fn2}\alpha }\\ + & \cdots \\ + & 2C_{fnN}\cdot err_{+N}\cdot \cosh\left(C_{fnN}\alpha \right)-C_{fnN}\cdot \Omega_{+N}\cdot e^{-C_{fnN}\alpha }\\ + & 2C_{fp}\cdot err_{-}\cdot \cosh\left(C_{fp}\alpha \right)-C_{fp}\cdot \Omega_{-}\cdot e^{-C_{fp}\alpha }=0 \end{array}$$
